# Thirst for Intention? Grasping a Glass Is a Thirst-Controlled Action

**DOI:** 10.3389/fpsyg.2019.01248

**Published:** 2019-06-04

**Authors:** Patrice Revol, Sarah Collette, Zoe Boulot, Alexandre Foncelle, Chiharu Niki, David Thura, Akila Imai, Sophie Jacquin-Courtois, Michel Cabanac, François Osiurak, Yves Rossetti

**Affiliations:** ^1^ Plate-forme “Mouvement et Handicap,” Hôpital Henry-Gabrielle, Hospices Civils de Lyon, Saint-Genis-Laval, France; ^2^ Inserm UMR-S 1028, CNRS UMR 5292, ImpAct, Centre de Recherche en Neurosciences de Lyon, Université Lyon 1, Bron, France; ^3^ Tokyo Women’s Medical University, Shinjuku, Japan; ^4^ Department of Psychology, Faculty of Arts, Shinshu University, Nagano, Japan; ^5^ Faculté de Médecine, Université Laval, Quebec, QC, Canada; ^6^ Laboratoire d’Etude des Mécanismes Cognitifs, Université de Lyon, Bron, France; ^7^ Institut Universitaire de France, Paris, France

**Keywords:** human, intention, psychomotor performance, kinematics, motivation

## Abstract

Every day and every hour, we feel we perform numerous voluntary actions, i.e., actions under the control of our will. Individual’s ability to initiate goal-directed movement is classically described as a hierarchical motor organization, from an intentional module, mostly considered as a black box, to muscular activity supporting action execution. The general focus is usually set on the triggering of action by intention, which is assumed to be the only entry to the action cascade, rather than on the preceding formation of intentions. If intentions play a key role in the specification of movement kinematic parameters, it remains largely unknown whether unconscious cognitive processes may also affect action preparation and unfolding. Recently, a seemingly irrelevant variable, thirst, was shown to modulate a simple arbitrary action such as key-pressing. Thirsty individuals were shown to produce stronger motor inhibition in no-go trials when a glass of water was present. In the present experiment, we intended to explore whether motor inhibition operates not only upstream from the action cascade but may also affect the unfolding of reaching movements, i.e., at a lower-level control. Thirsty vs. non-thirsty control subjects were asked to reach and grasp green (go trial) or red glasses (no-go trial) filled with either water or transparent gel wax with a central candlewick. Thirsty subjects were faster to initiate actions toward the water glasses. They also exhibited an earlier maximal grip aperture and a global reduction of movement time which was mostly explained by a shortening of deceleration time. The deceleration phase was correlated with individual’s thirst rating. In addition, no-go trial toward a glass of water tended to inhibit the next movement toward a glass filled with gel wax. Thus, our results show that an unintentional influence of an internal state can reorganize voluntary action structure not only at the decision-making level but also at the level of motor control. Although subjects explicitly paid more attention and were more cautious to glasses filled with water, they reported no explicit sensation of an increased urge to grasp it, further suggesting that these effects are controlled by covert mechanisms.

## Introduction

The most acknowledged feature of voluntary actions is that they are internally generated, unlike reactions to external stimuli. Descartes conceived voluntary action as resulting from the effects of transcendental spirits on animal spirits, which, in turn, would blow into nerves to inflate muscles and produce forces. This ancient view fits well with the individual subjective experience accompanying action: I initially need to think that I want to grasp an object, and then machinery makes my hand move. We are all aware that we do not have to convert directly our intentions into individual patterns of muscle commands. Modern neurophysiological models of action follow pretty much the same scheme. Classical cognitive neuroscience views of action organization involve an original intention module wherefrom a cascade of process takes place and terminates as muscle activity exerting forces in the environment (Jeannerod, 1986, 1988). For example, Marc Jeannerod’s depiction of action distinguishes four major steps: intention, planning, programming, and execution (Jeannerod, 1986). This functional structure may be associated with corresponding neural structures, e.g., pre-frontal cortex, premotor cortex, supplementary motor area, and primary motor cortex. Most logically, such representations of the action organization provide descriptions of action steps whose quality of details is inversely proportional to the hierarchical level. We understand better the muscles, spinal cord, and primary motor cortex than our intentions. As a matter of fact, the most refine knowledge is available about neural circuits, neuronal activities, and connectivity for the execution level of action associated with primary motor cortex and spinal cord. Planning and programing phases of action preparation have been distinguished on a functional basis and their precise neuroanatomical substrates and neuronal mechanisms remain to be further investigated (e.g., Cisek, 2007). In addition, the functional as well as anatomical boundaries between programming and planning of action remain somehow speculative (e.g., Thura and Cisek, 2014, 2016). Most current models of action do not actually address the nature of intentions and they simply refer to this concept as the starting point of the voluntary action process without further considerations. Recent studies shown that intentional actions can be modulated by decision-making and motor programming (Brass and Haggard, 2008; Becchio et al., 2014). Intention can be defined as a specific thought that you will be making a movement (Matsuhashi and Hallett, 2008). Intention is usually associated with the prefrontal cortex functions (e.g., Lhermitte, 1986; Jeannerod, 1990), although a broader circuitry can be evoked (Haggard, 2008; Shadlen et al., 2008; Andersen and Gui, 2009; Freedman and Assad, 2011).

Experimentally, it is harder to study the positive than the negative manifestations of intention, i.e., consequences of its inefficiency in healthy controls (e.g., Pisella et al., 2000; Rossetti and Pisella; 2002) or neurological patients (Lhermitte, 1986; Niki et al., 2009, 2019). Lack, reduction or impairment of intentional control of action is usually manifested in the form of reduced inhibition of automatic responses (Lhermitte, 1986; Rossetti and Pisella, 2003; Niki et al., 2009). In the field of cognitive neuroscience, most studies about the positive manifestations of intention have addressed the issue of the timing of intention with respect to action execution (Libet, 2002; Salvaris and Haggard, 2014), e.g., at the level of extremely simple motor command such as moving one finger (Libet, 1983; Frith and Haggard, 2018). The identification of neural activities that seem to precede the estimated timing of explicit feeling of intention has been used to question the very reality of intentions and of free-will (e.g., Wegner, 2003; Ebert and Wegner, 2011). However, other authors challenged that awareness of willing and acting are not always causally related but can be double dissociated (e.g., Spence, 2009; Jeannerod, 2012), and the very significance of brain signals associated with movement onset has been challenged (e.g., Schurger et al., 2012, 2016). In addition, it is questionable whether the validity of action models dealing with only a few hundreds of milliseconds can extrapolated to more complex everyday actions (Pisella et al., 2000; Jeannerod, 2012). One of the most puzzling questions raised by the cognitive neuroscience approach to intention is about its status in terms of cause or consequence (e.g., Jeannerod, 2012; Frith and Haggard, 2018).

Beyond this hierarchical cascade model of action, another important field of investigation focused on the roots of intention. Following the idea that cognition is mostly composed of unconscious processes (Kihlstrom, 1987), it has been argued that intention cannot be conceived as a simple step in a serial model but should be embedded in a broader context (Custers and Aarts, 2010; Bode et al., 2014). The crucial feature of this view is that unconscious needs may give rise to intentional actions. It has been long known that physiological parameters such as thirst or hunger can alter perception (e.g., Cabanac et al., 1971; Balasko and Cabanac, 1998; Bhalla and Proffitt, 2000) and modulate motivations. Recently, Summerside et al. (2018) evidenced that human reaching was more vigorous when there was a greater expectancy of reward. One important question is about whether such perceptual effect may affect actions prior to or at their origins, i.e., intention and planning, or whether they may alter directly lower levels of the action cascade. The idea that pleasure is “the common currency” for the brain to compare incommensurable activities and rewards (Cabanac, 1992) implies that such weighing process takes place at least before the decision for action is made and even before the action is selected, hence at the motivational level (e.g., Balasko and Cabanac, 1998), i.e., within the roots of intention. However, one may expect unintentional drives to also directly affect further stages of action realization.

Measurement of these unintentional urges to act can be measured by the strength of inhibitory control required to keep intentional control of action. For example, motivational urges can be measured against unpleasant conditions: increasing hunger or palatability of food accessible in a cold place will increase the time spent by rats in this uncomfortable environment (Balasko and Cabanac, 1998). As another example, no-go trials have been introduced to explore this inhibitory control. In a simple and clever experiment, Veling and Aarts (2011) required subjects to perform key-presses in response to visual stimuli coupled with images of a glass of water or other objects. Following a no-go trial, thirsty subjects were found to exhibit enhanced inhibitory control which manifested itself as a lengthening of the reaction time in the following go trial. This indirect argument suggested that being thirsty would raise the need to inhibit reactions in no-go trials where a glass of water was displayed, i.e., the impulsion to act was stronger because thirst produced an unconscious motivation to act (for somewhat similar results on attentional interference, see Mazzietti et al., 2014). If thirst is capable of inducing such effect on the reaction time of the next trial in the sequence, it is possible that direct effects of thirst can be measured during the simple reach-to-grasp action toward a glass of water.

In our experiment, we asked subjects to reach-and-grasp a glass in front of them in order to study the direct effects of thirst on the movement latency and kinematics. Specifically, an obvious prediction is that thirst may shorten the duration needed to access water, i.e., alter reaction time and/or movement time. In addition, our strong prediction is that kinematic landmarks reflecting the structure of either the reach or the grasp component may be altered by thirst, which would provide arguments for an effect of unintentional motor impulses beyond the level of decision-making. Moreover, we imported the go/no-go paradigm from Veling and Aarts (2011) in order to measure the indirect effects of inhibiting movements toward a glass of water on the following movement in the sequence, which may be more marked for grasping a glass than for key-pressing movements.

Although previous studies used simple finger lifting or key-pressing to address these issues, we selected grasping movements for two main reasons. First, these movements are highly ecological and the most performed action in everyday life. Second, the kinematic analysis of reach-to-grasp action is rich and offers access to numerous individual parameters that reflect several functional or pathological processes (Jeannerod, 1981; Jeannerod et al., 1998; Weiss and Jeannerod, 1998; Castiello, 2005). Although these processes are uniquely combined to achieve prehension of external objects, they can also be dissociated by experimental conditions or brain lesions (Jeannerod, 1986; Wing et al., 1986; Paulignan et al., 1991; Jeannerod et al., 1995; Milner et al., 2001; Gréa et al., 2002). Therefore, grasping movements provide an optimal tool for studying the potential effects of thirst on the organization of action. Specifically, we predicted that the kinematics of reach-to-grasp movements aimed toward glasses containing beverages vs. non-drinkable products are differentially affected by thirst.

## Materials and Methods

Twenty healthy subjects from 19 to 32 years old (12 females and 8 males) participated in this study. They were randomly distributed into two groups. In the first one (thirsty group, 6 females and 4 males), subjects were asked to come to the laboratory following a period of at least 6 h without drinking. In the other (control group, 6 females and 4 males), no limitation was required. Upon arrival, subjects were asked to rate their thirst on a visual analogic scale ranging from 0 to 10 (0: not thirsty; to 10: very thirsty). Individuals enrolled in the control group were required to drink a glass of water before the preparation of the experiment. Thirst assessment was repeated immediately at the end of the experiment in order to more closely reflect thirst during the experiment. Following this last rating, thirsty subjects were offered a glass of water. All subjects were right handed as assessed with the Edinburg Handedness Inventory (Oldfield, 1971). All participants were naïve as to the purpose of the experiment and gave informed written consent in accordance with the declaration of Helsinki. The Inserm Ethic Committee reviewed and approved this study in healthy participants.

The subject is seated in a comfortable chair in front of the experimental set-up with his head in a chin rest. It prevents both head movement during the experiment and the vision of another glass in the current trial.

**Figure 1:**
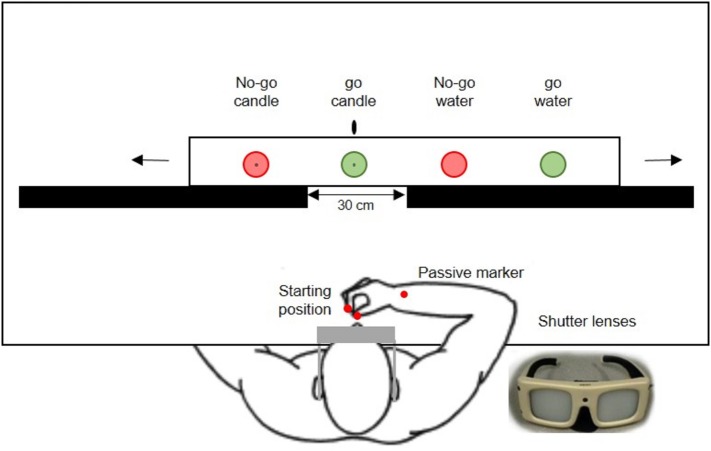
Drawing of the experimental set-up. The subject wears shutter goggles which become transparent to indicate trial onset, allowing the subject to reach and grasp green glasses. Red glasses are used for no-go trials. One glass of each color is filled with water, whereas the other one is filled with transparent gel wax and includes a central candlewick.

The experimental set-up (Figure 1) included a window (30 cm) aligned with the sagittal plane, where a glass was seen, surrounded by two wooden screens. Behind these screens, there was a wooden board with four different glasses, which slides on a tray thanks to a handle used by the experimenter during the rest period between trials, i.e., while the subjects’ vision was occluded. The displacement of the board between trials made little noise. In order to prevent a potential bias, such as target prediction, the experimenter moved the board two or three times on the left and right before placing the selected glass. Markings both on the table and on the wooden board allowed to place the selected glass in the sagittal plane at the center of the window, and the other three were occluded by the screens. Moreover, the subject wore a pair of shutter goggles (Translucent Technologies, Toronto, ON) with liquid-crystal shutter lenses, which were connected with the pilot computer with custom software used to control the vision of the stimulus. The liquid-crystals were opaque when the subject’s hand was on the starting position, which also allowed the experimenter to place the selected glass in the center unbeknownst to the subject. Then they became transparent at trial onset, before the subject could see the glass and initiates her/his response (or not).

Subjects had to grasp the glass presented in the sagittal plane at a distance of 30 cm only if it was a go trial, i.e., a green glass. They were asked to naturally lift it and replace it in its initial position. Each subject performed a unique session with 96 trials, which lasted approximately 30 min. They knew that glasses were filled either with water or gel wax. Different glasses were divided in two categories: go trial with green glasses filled with either water (water glass) or transparent gel wax with a central candlewick candle (candle glass) and no-go trial with red glasses filled with either water or transparent gel wax with a central candlewick. The weight of the glasses was similar 120 g (SEM: 0.40 g) as well as the water and gel wax levels (5.0 cm). In a session, the four glasses were presented successively using the six permutations of four items. Each glass was presented 24 times in a pseudo-random order and the same glass was never presented in two consecutive trials. Using this trial design, there was an equal probability for each glass in each position. Before data acquisition subjects were asked about their color vision, a small training session (four trials) with green and red empty glasses was tested in order to familiarize the subject with the procedure. Subjects had to reach and grasp glasses with the same instruction as during the experiment: go response to green glasses and no-go responses to red glasses.

Hand movements were recorded using a 3D motion capture system (Motion Analysis®) composed of nine infrared stroboscopes at a sample rate of 200 Hz. Three infrared passive reflecting markers were placed in specified anatomical location using bone palpation: index and thumbnails, styloid radial of the wrist. After recording and 3D reconstruction, the position of each marker was filtered with a Butterworth low-band pass filter, with a cut-off frequency of 6 Hz. Then, from the markers’ spatial position, movement parameters were computed using a homemade software Handimain developed by Inserm ImpAct engineers. Thus, movement initiation, i.e., Reaction Time (RT) and reaching parameters were computed: Time Task (TT) to perform the movement (TT = RT + MT), i.e., the time between go-signal and the end of the movement i.e., the glass grasp, Movement Time (MT), Acceleration Phase (AP), and Deceleration Phase (DP). For the grasping parameters, Maximal Grip Aperture (MGA), Time to Maximal Grip Aperture (TMGA) and Finger Closure Time (FCT = MT − TMGA) were also computed. These movement parameters were determined in a semi-automatic procedure with trial-by-trial validation by one expert experimenter (PR).

Statistical analysis was conducted with Statistica® 13 using repeated measure ANOVA with two main factor, group (thirsty or control) and glass (water or candle). Unilateral *t* tests were performed as previous data showed that thirsty subject reacted faster than controls (Veling and Aarts, 2011). Additionally, according to previous results (Veling and Aarts, 2011), it could be postulated that motor inhibition occurring in no-go trial spread to the following trial. To test this specific hypothesis planned comparisons were used to compare inhibition effect by candle no-go or water no-go trials on the following go trials with candle glasses, both in the control and the thirsty groups.

## Results

Subjects debriefing revealed that none of the subjects reported an increased urge to grasp glasses of water or faster movement production. In the two groups, subjects (six thirsty and seven controls) instead reported that they paid more attention to grasp the glasses filled with water. Interestingly, no participant drunk or attempted to drink from the experimental glasses filled with water during the course of the experiment although there was no interdiction nor authorization.

The subjective thirst rating was compared between the two groups. As expected, thirst was significantly higher in the thirsty group both on arrival [5.75 vs. 3.15, Student test *t*(1,15) = 2.78, *p* = 0.0144] and at the end of the experiment [7.15 vs. 2.55, Student test *t*(1,15) = 6.94, *p* = 0.000005].

**Figure 2:**
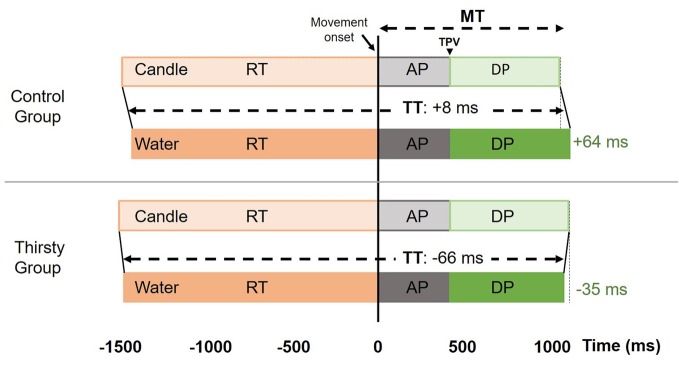
Kinematic parameters were illustrated for both groups (control vs. thirsty) and both glasses (candle vs. water) in go trials. In the control group, the TT was similar for both glasses with an increase of DP when the glass is filled with water compared with candle. In the thirsty group, the glass filled with candle leads to a significant reduction of TT due to both a reduction of RT combined with a reduction of DP. RT, Reaction time; MT, Movement time; TPV, Time to peak velocity; AP, Acceleration phase; DP, Deceleration phase, TT, Time task to perform the movement, from go-signal to glass grasp. All movements were aligned with the movement onset.

As expected in healthy individuals and without time pressure, no subjects initiated a movement in the no-go trials. Considering the go trials, kinematic data were summarized in Table 1 and Figure 2. The most comprehensive kinematic parameter is the time to perform the task (TT) that corresponds to the time between the go signal and the end of the movement, i.e., the sum of reaction time and movement time. The ANOVA did not reveal any main factor effect [*F*(1,18) = 0.06, *p* = 0.41, $\eta_{p}^{2}$ = 0.003; *F*(1,18) = 2.10, *p* = 0.08, $\eta_{p}^{2}$ = 0.10 for group and glass factors], but an interaction effect was found [*F*(1,18) = 3.10, *p* < 0.05, $\eta_{p}^{2}$ = 0.147]. In the control group, TT is very close for the two glasses, while in the thirsty group, a shortening of 66 ms (Figure 2) occurred when the glass was filled with water. Further analyses enabled us to determine whether this effect was due to a reduction of reaction time or movement time or both.

**Table 1:** Mean and SD for each kinematic parameter for control and thirsty groups when they grasped glasses filled with candle or water.

			TT (ms)	RT (ms)	MT (ms)	AP (ms)	DP (ms)	MGA (mm)	T MGA (ms)	FCT (ms)
Controls (*n* = 10)	Candle	Mean	2585.7	1514.0	1071.7	429.2	642.5	97.1	687.5	384.2
		SD	322	187	220	89	159	9	159	28
	Water	Mean	2592.1	1456.1	1136.0	430.0	706.0	96.7	744.5	391.5
		SD	333	184	186	90	121	9	126	29
Thirsty (*n* =10)	Candle	Mean	2662.0	1531.9	1130.1	429.1	701.0	90.5	716.2	413.9
		SD	421	219	215	85	145	7	138	28
	Water	Mean	2596.2	1501.2	1095.1	424.3	670.8	88.9	673.0	422.1
		SD	403	195	219	76	170	5	151	29

TT, Time task to perform the movement from go-signal to the end of reach-to-grasp the glass; RT, Reaction time; MT, Movement time; AP, Acceleration phase, DP, Deceleration phase; MGA, Maximal grip aperture; and T MGA, Time to maximal grip aperture; FCT, Finger closure time.

As for the initiation of movement, the ANOVA performed on RT failed to reveal a significant group effect [*F*(1,18) = 0.14, *p* = 0.36, $\eta_{p}^{2}$ = 0.01] but a significant glass effect was obtained [*F*(1,18) = 4.83, *p* = 0.0414, $\eta_{p}^{2}$ = 0.21]. The movement started 44 ms earlier when the glass was filled with water. No interaction was evidenced [*F*(1,18) = 0.45, *p* = 0.26, $\eta_{p}^{2}$ = 0.025]. For the MT parameter, neither group effect [*F*(1,18) = 0.01, *p* = 0.46, $\eta_{p}^{2}$ = 0.0005] nor glass effect [*F*(1,18) = 0.40, *p* = 0.27, $\eta_{p}^{2}$ = 0.02] were found, while there was a significant interaction [*F*(1,18) = 4.63, *p* = 0.0452, $\eta_{p}^{2}$ = 0.20]. Conforming to speed-accuracy trade-off predictions (Marteniuk et al., 1987; Wallace and Weeks, 1988; Paulignan et al., 1991), the control group took 64 ms more to reach and grasp the glass filled with water. Conversely, in the thirsty group, it took 35 ms less, suggesting that thirst induced a faster movement toward water glasses (Figure 2), which corresponds to a relative difference of about as much as 100 ms shortening.

**Figure 3:**
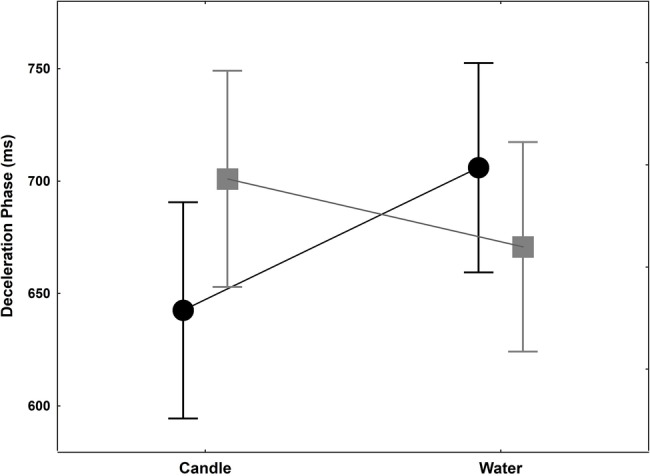
Deceleration phase (ms) +/− SEM for controls (black circle) and thirsty groups (gray square) when they reached and grasped the candle or water glass. This phase is 30 ms shorter when thirsty group have to grasp the glass filled with water while it is lengthening in control group.

Time to peak velocity failed to reveal any statistical difference for the groups main factor [*F*(1,18) = 0.06, *p* = 0.47, $\eta_{p}^{2}$ = 0.0003; *F*(1,18) = 0.09, *p* = 0.38, $\eta_{p}^{2}$ = 0.01]. No interaction was found [*F*(1,18) = 0.20, *p* = 0.33, $\eta_{p}^{2}$ = 0.11]. The deceleration phase is the delay between time to peak velocity and the end of the movement. No significant effect for group and glass factors was evidenced [*F*(1,18) = 0.03, *p* = 0.43, $\eta_{p}^{2}$ = 0.002; *F*(1,18) = 0.64, *p* = 0.22, $\eta_{p}^{2}$ = 0.03, respectively]. A significant interaction was found [*F*(1,18) = 5.10, *p* = 0.0367, $\eta_{p}^{2}$ = 0.22] with a decrease of 30 ms (out of about 35 ms difference in MT) in the thirsty group when they had to reach and grasp a glass filled with water, while an increase of 63.5 ms was observed in the control group (Figure 3). Thus, the shortening of movement time in the thirsty group is due to a shortening of deceleration phase, with a differential value of as much as 94 ms less than in the control group.

For the grasping parameters, the ANOVA performed on the maximal grip aperture revealed a group effect [*F*(1,18) = 4.84, *p* = 0.0411, $\eta_{p}^{2}$ = 0.21], with the control group opening the pinch 7 mm wider. The contents of the glass were not significant [*F*(1,18) = 1.52, *p* = 0.12, $\eta_{p}^{2}$ = 0.08] nor the interaction [*F*(1,18) = 0.53, *p* = 0.24, $\eta_{p}^{2}$ = 0.03]. Time to maximal grip aperture, i.e., the opening time, failed to show main effects [*F*(1,18) = 0.13, *p* = 0.37, $\eta_{p}^{2}$ = 0.007 and *F*(1,18) = 0.09, *p* = 0.39, $\eta_{p}^{2}$ = 0.01 for group and glass factors]. A significant interaction was found [*F*(1,18) = 4.79, *p* = 0.0422, $\eta_{p}^{2}$ = 0.21], with a MGA occurring 43 ms earlier in the thirsty group, coherent with the shortening of the movement time and the deceleration phase when the glass is filled with water. Strikingly, this reduction is even more substantial than the reduction of total movement time (−35 ms). By contrast, it was 53 ms later in the control group (Table 1), i.e., a relative difference of nearly 100 ms between the two groups (i.e., by the same amount as TT and DT). Finally, the Finger Closure Time (FCT = TM − TMGA) failed to reveal any significant main effect [*F*(1,18) = 0.60, *p* = 0.45, $\eta_{p}^{2}$ = 0.03; *F*(1,18) = 0.75, *p* = 0.40, $\eta_{p}^{2}$ = 0.04 for group and glass factors], nor interaction [*F*(1,18) = 0.002, *p* = 0.96, $\eta_{p}^{2}$ = 0.0001]. However, in the thirsty group, a 30 ms lengthening was found for the glass filled either with water or with candle compared with controls.

**Figure 4:**
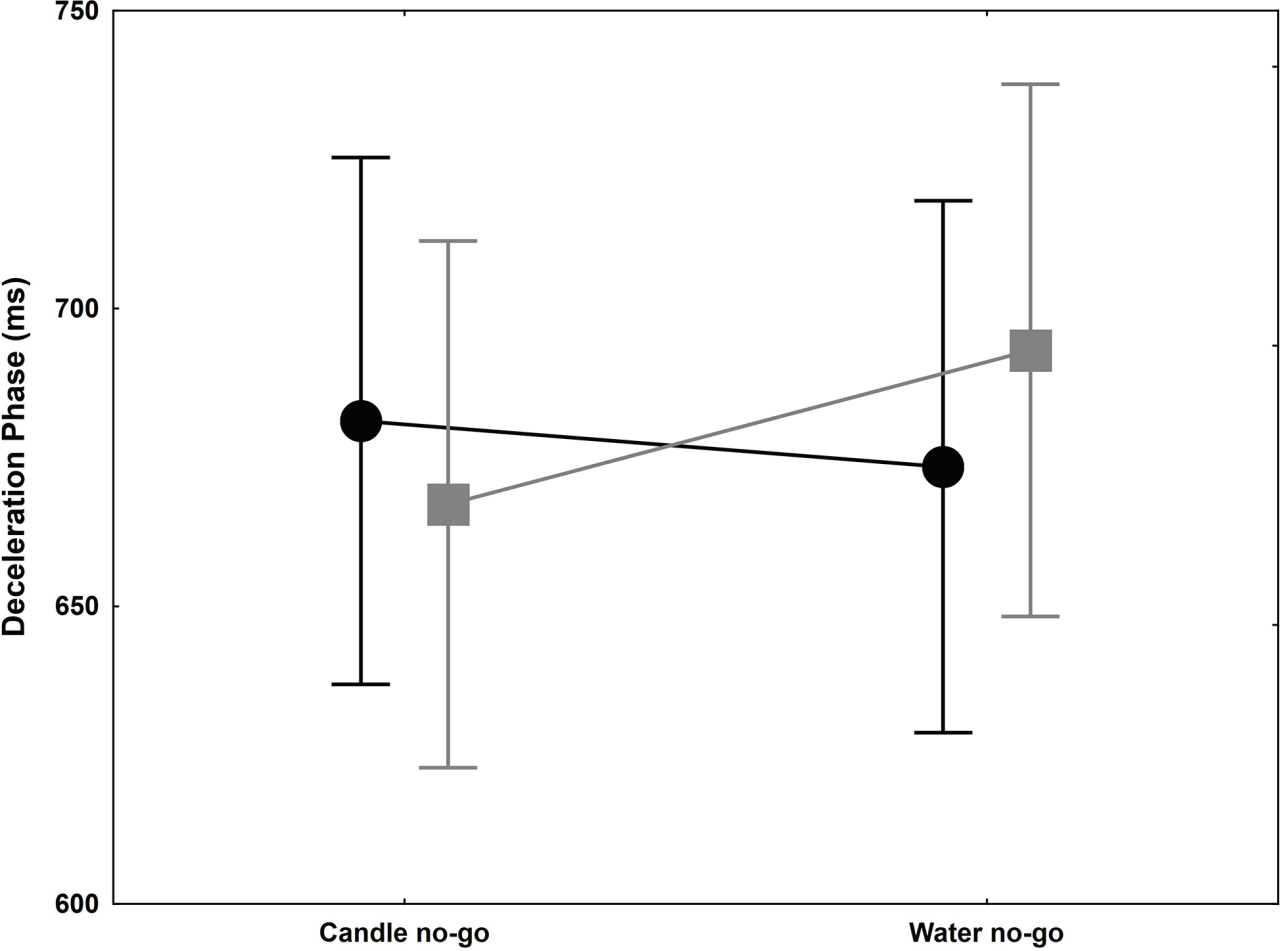
Deceleration phase (ms) +/− SEM for control (black circle) and thirsty groups (gray square) when they reached and grasped the candle glass and precluded either by a candle no-go or water no-go trial. This phase is slightly shortened in the control group while it was significantly lengthened by 25 ms in the thirsty group.

Veling and Aarts (2011) showed that motor inhibition activated by thirsty subjects in a no-go trial toward a glass of water spread to the following trial. In order to confirm this finding in a more ecological task, we compare the inhibition effect by candle no-go or water no-go, i.e., red glasses, on the following go trial with transparent gel wax. Only one parameter yielded to significant result: DP (Figure 4). The ANOVA on DP failed to reveal any significant group and glass main effects [*F*(1,18) = 0.002, *p* = 0.48, $\eta_{p}^{2}$ = 0.0001; *F*(1,18) = 0.75, *p* = 0.20, $\eta_{p}^{2}$ = 0.04]. A tendency can be observed for the interaction [*F*(1,18) = 2.56, *p* = 0.06, $\eta_{p}^{2}$ = 0.12], and a significant 25 ms lengthening of the DP is evidenced in water no-go in the thirsty group [planned comparison, *F*(1,18) = 5.04, *p* = 0.0376], while no difference was evidenced in the control group [planned comparison, *F*(1,18) = 0.27, *p* = 0.60]. These results were in agreement with Veling and Aarts (2011), suggesting a motion slowness in go trial with transparent gel wax even if the delay between trials was longer in our experiments (15 vs. 1 s). For the RT, no main effect was significant [*F*(1,18) = 0.13, *p* = 0.37, $\eta_{p}^{2}$ = 0.01; *F*(1,18) = 0.01, *p* = 0.46, $\eta_{p}^{2}$ = 0.001 for group and glass factors] and no significant interaction [F(1,18) = 1.63, *p* = 0.11, $\eta_{p}^{2}$ = 0.08]. However, there is a slight but not significant lengthening in the thirsty group following a water no-go trial, which correspond to a relative difference with controls of about as much as 62 ms. This result confirms a tendency to initiate slower the next trial (Veling and Aarts, 2011).

**Figure 5:**
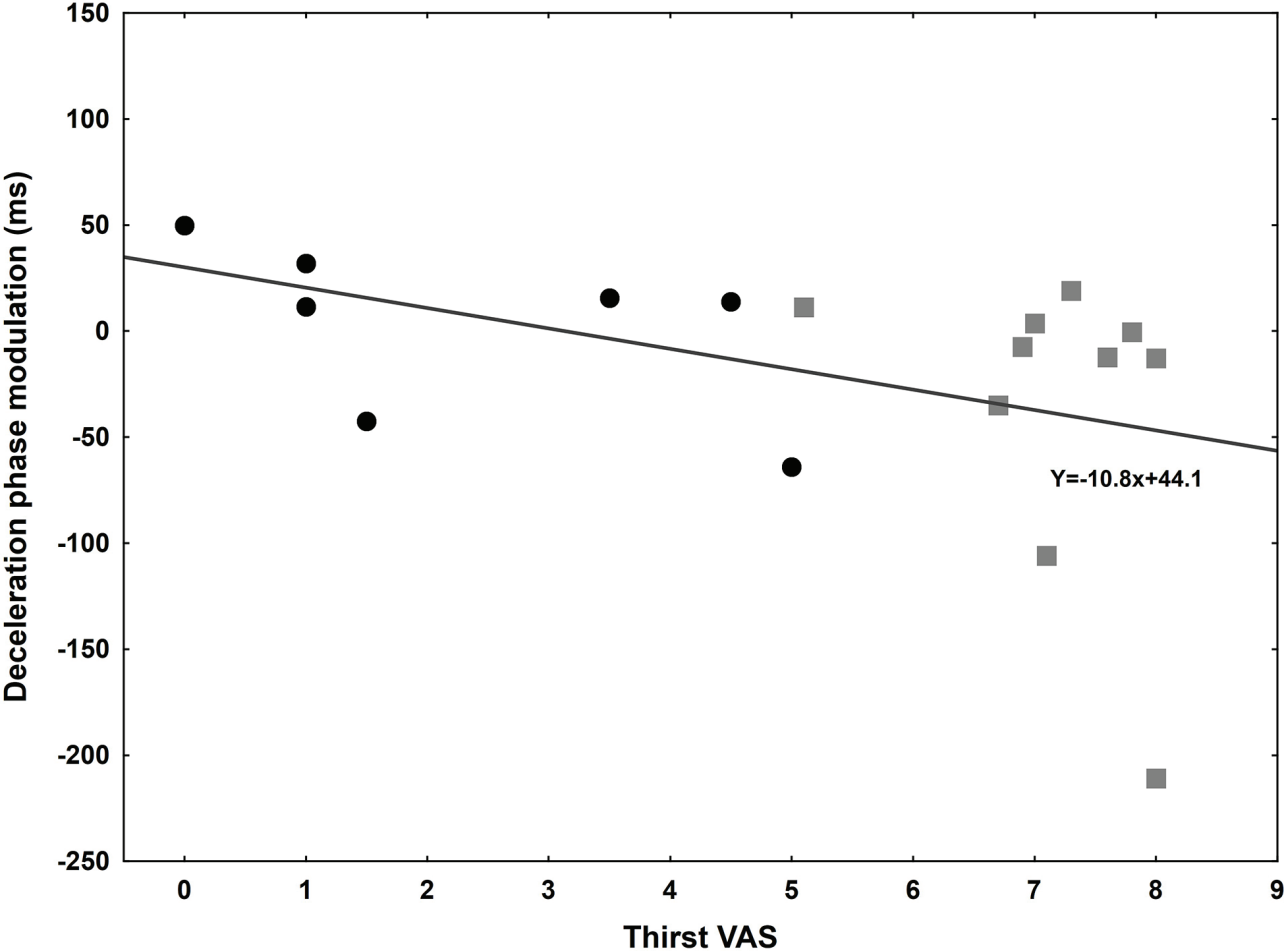
Correlation between Thirst’s Visual Analogical Scale at the end of the experiment and the deceleration phase modulation (ms) between control (black circle) and thirsty (gray square) groups. The more important the thirst is; the shortened this phase is for a goal-directed movement towards the glass filled with water.

Using a visual analogical scale, subjects were asked to report their thirst before and after the experiment in 17 out of 20 subjects. In the thirsty group, the score increased at the end of the experiment (from 5.75 to 7.15), while it was not the case in the control group who drank before the onset of the experiment (from 3.15 to 2.55). Moreover, the score at the end of the experiment was used to investigate correlations with the temporal difference between water and candle glasses for several kinematic parameters (Table 2). The regression analysis was only significant for deceleration phase [*F*(1,15) = 4.75, *p* = 0.0457], suggesting a link between thirst and movement time reduction, i.e., the increased thirst predicts a shortening of DT when movements are performed toward the glass filled with water (Figure 5).

**Table 2:** Statistical correlations between Visual Analogical Scale estimates of thirst at the end of the experiment and main kinematic parameters.

	*F*	*p*	*r^2^*
TT	*F*(1,15) = 1.60	*p* = 0.13	0.12
MT	*F*(1,15) = 2.91	*p* = 0.10	0.16
AP	*F*(1,15) = 0.68	*p* = 0.42	0.01
DP	*F*(1,15) = 4.75	*p* = 0.0457	0.24

## Discussion

Several main findings shall be discussed below. First, thirsty subjects were faster to reach-and-grasp the glass of water. Second, alterations of kinematic parameters in the thirsty group suggest that a modification of the reach-to-grasp action structure resulted from thirst. Third, our results tend to replicate previous results about inhibition of action in thirsty subjects in the presence of a glass of water.

First, our experiment enabled us to contrast movements performed by thirsty and by control individuals, in whom speed-accuracy trade-off predicts that grasping the glass of water should increase MT in order to cope with the increased risk that was subjectively reported by several subjects from the two groups. As a matter of fact, a total task time reduction of 66 ms in the thirsty group whereas the control group managed to keep the total task duration constant (Table 1). In the control group, a shortening of RT compensated the lengthening of MT. The control group actually exhibited a longer MT for the glass of water (+64 ms for the glass of water), whereas the thirsty group showed a shortening of MT by 35 ms for the glass of water vs. the candle glass. Therefore, the thirsty group reached relatively faster the glass of water (about 100 ms). These results are in agreement with previous studies showing that movements were faster when there is a greater reward at stake (Reppert et al., 2015; Summerside et al., 2018), linking motivation and action. Modulations of the RT appear to compensate for the slowing down of movement in the control group. A main glass effect was found with a significant lengthening of the initiation phase for the candle glass. The RT in the control group was 42 ms shorter when having to reach-and-grasp the glass of water, such that their total task time remained fairly constant. In contrast, the thirsty group was 31 ms shorter to initiate their movements toward the glass of water, which instead increased the difference in total task time between the two groups. The important MT modification and relative changes between the two groups suggest that the controlled variable was the MT—presumably in relation to speed-accuracy trade-off—whereas the RT can be modulated in a compensatory manner in the control group. This result provides evidence that the covariation between RT and MT can be flexible, and that there seem to be limitations to the hypothesis that a common mechanism governs speed-accuracy trade-off adjustments during decision-making and movement execution (Thura and Cisek, 2016). This also reveals that action elements may be altered in non-proportional way when a global movement shortening is observed.

Second, and beyond the expected effect on the task duration, the question also arises about whether such variable (RT and MT) may affect the organizational structure of the action. Most previous studies about unconscious volition assessed the role of unconscious processes in the inhibition, release or choice of a specific behavior (e.g., Veling and Aarts, 2011). The current study investigated whether unconscious processes would affect the unfolding of the action itself. When it comes to the internal structure of the movement, we found a non-significant decrease of acceleration time in the thirsty group. This contrasts with the large difference found between the two groups for deceleration time. In the control group, subjects were 44 ms slower to reach for the glass of water. As a matter of fact, an increased need for accuracy is known to result mainly in a lengthening of the deceleration phase, i.e., when visual feedback is available. This has been repeatedly shown for pointing (e.g., Fitts, 1954) and applies to grasping (Marteniuk et al., 1987, 1990). In sharp contrast with this constraint, the thirsty subjects exhibited a significant shortening of deceleration time when reaching toward the glass of water (−30 ms out of the −35 ms decrease in MT). This suggests that the ultimately controlled variable during the reach-to-grasp was the deceleration phase whereas the acceleration phase remained constant. An additional argument lies in the finding that individual thirst rating constitutes a predictive factor for deceleration time shortening.

In addition, alterations of the grasping component kinematics were also observed. Among the parameters that affect reach-to-grasp kinematics, there are obviously the target object size and distance (Wing et al., 1986; Castiello, 2005; Rand et al., 2006) but also the action’s end-goal (Ansuini et al., 2008; Crajé et al., 2011; Sartori et al., 2011) and the social context (Georgiou et al., 2007). Initial reports focused on the apparent temporal linking between the transport and the prehension parameters (Jeannerod, 1990; Marteniuk et al., 1990; Paulignan et al., 1991; Jeannerod et al., 1995). The effect of varying the speed of the reach-to-grasp on the grasping component of the action has also been specifically investigated (Wing et al., 1986; Rand et al., 2006; Fukui and Inui, 2015). It has been shown that the maximal grip aperture is crucially depending on the duration of the transport, more than on the movement velocity (Wallace and Weeks, 1988). Consistent with the speed accuracy trade-off, a minor increase of grip opening time and of closing time were observed in our control group for the glass of water. Conversely a 33 ms shortening of grip opening time was found in the thirsty group when they reached toward the glass of water, which magnitude seems sufficient to explain their movement time effect (−35 ms). However, the thirsty group did not show a shortening of the grip closure time, which may have jeopardized the successful grasping of the glass.

One may have predicted that thirsty subjects may open their grip smaller if their increased need for water made their perception of the water glass smaller. A group effect was found on maximal grip aperture with a 7 mm smaller pinch opening in the thirsty group. Several studies showed that it is the distance travelled by the wrist after maximal aperture that constituted the controlled variable (Wang and Stelmach, 1998, 2001), although extreme speed difference comparison revealed that grip aperture can also be affected by movement speed (Rand et al., 2006). However, only marginal variations of maximal grip aperture were observed when “comfortable” vs. “as fast as possible” movements were performed showing the high stability of this variable (Wang and Stelmach, 1998). When important variations of movement were investigated, it was found that the effect of movement time on grip aperture affected only the grip closure time (Rand et al., 2006), i.e., mostly during the deceleration phase. However, this effect was significantly observed only for speeded movements (about 500 ms) lasting much less than the 1,100 ms range observed in our experiment. Even though maximal grip aperture was shown to increase when movement speed increases in order to compensate the transport phase loss in accuracy, in the present study, the modulation of MT may not be of sufficient magnitude to reveal reliable effects on MGA. Previous studies have revealed that illusory (Kopiske et al., 2016) or irrelevant cues may alter the size of the grip formation. For example, Gentilucci et al. (2000) showed that grasping an object was affected by words printed on the object. For example, maximal grip aperture was increased when the word “large” was on the object. Moreover, lifting action was higher when the verb “lift” was used (Gentilucci, 2003). In a similar vein, Tubaldi et al. (2008) showed that smelling the flavor of a large fruit while grasping a small one resulted in a widening of the finger grip. These examples reveal that several entries can affect motor production and in the presence of incongruent information provided by two independent canals the influence of the two can be observed in the motor output. The use of words typically calls for an intervention of highly cognitive functions that would affect the intentional or the planning phase of action, i.e., the highest levels of the action cascade.

Third, from the point of view of cognitive neuroscience of action, intention lies at the origin of a cascade leading to coherent muscle activations (e.g., Jeannerod, 2009). Motivational factors are usually acknowledged in as much as they feed into intention building processes. Therefore, their conscious and unconscious influences are considered to affect action only prior to or right at its origin by increasing the probability for a given behavior to take place (e.g., Balasko and Cabanac, 1998). Another way these influences can be manifested is by modulating time allocated to perform the action, altering the urge to act or obtain a given reward. There is no need for experimentations to demonstrate that somebody thirsty is more likely to exhibit a drinking behavior than somebody who just had a glass of water (although this may not be true for all beverages). Conversely, when thirsty individuals have to inhibit a movement while a glass of water is displayed on a computer screen, they exhibit a sustained inhibition affecting the next movement they have to perform, which provides a measure of the increased drive toward water (Veling and Aarts, 2011). Therefore, physiological variables like thirst can contribute to the decision-making stage of action. Although marginally significant, our results tend to confirm this result: our thirsty subjects exhibited an increased RT (+28 ms) in trials toward a candle glass when the preceding trial in the sequence was a no-go trial toward a glass of water, whereas the control group showed a decreased (−34 ms). The fact that the effect found here seems to be of less magnitude than in Veling and Aarts (2011) is likely to be attributed to a much longer inter-trial interval in our experiment (about 15 s) as compared to the original study (1 s).

The main question raised in this study was about whether kinematic analysis could reveal structural changes in movement produced by thirsty subjects reaching for a glass of water. The above-discussed kinematic alterations provide support for this hypothesis. It should be emphasized that participants from our two groups reported that aiming for the glass of water required more care. In addition, participants were never offered to drink from the experimental glasses, and spontaneously assumed that they should not drink from these glasses, as none of our subjects did. Given these elements, it appears quite unexpected that subjects who repeatedly reach-to-grasp glasses which contain either water or candle would perform different actions to these two goal objects which they would not differently use ultimately. According to the classical cognitive neuroscience view of action, they should simply grasp these laboratory objects repeatedly without being influenced by their slight differences, given that these apparent differences do by no mean imply that they may lead to quench their thirst. The perceptual relevance of water was therefore not associated with a behavioral relevance. In spite of this prediction, thirsty participants not only performed faster actions, but exhibited changes during the unfolding of their actions. Performing their action faster can be associated with an influence of thirst on the decision process leading to the initiation of action, but such effect was not observed here. However, the trend seen in our data set remains compatible with the results obtained by Veling and Aarts (2011). The fact that our subjects performed their actions faster toward the glasses of water and that this was mostly explained by a reduction of deceleration time and a shortening of grip opening time suggests that their actions structure was reorganized to enable the realization of faster reaches that would remain compatible with successful action outcome. The fact that not a single participant commented on shortening movement time or on the kinematic modifications and instead experienced being more careful when reaching to the glass of water further supports the idea that these effects must be ascribed to unconscious influence of thirst on action. Our results further support the idea that potentially rewarding targets unintentionally not only prepare (Veling and Aarts, 2011) but also configure actions. Therefore in addition to the motivational value of the water reward that prompts the motor system to react and increase the likelihood of a given behavior, our results point to a deeper, unintentional influence of the motivational drive on the structural organization of the motor action that is intentionally performed. Coming back to the classical view of cognitive neuroscience of action, our results suggest that unintentional impulses, although not necessarily resulting from unconscious feelings, may also affect the way actions are performed in addition to the engagement of action.

As a final note, it can also be stressed that our findings complete those obtained on the influence of intentions or physiological states on perception. Cabanac et al. (1971) first demonstrated that losing weight leads to a sustained appetence for sugar. For instance, people who are encumbered (e.g., wearing a heavy backpack), physically fatigued, or in declining health overestimate distances and hill’s slant (Bhalla and Proffitt, 1999, 2000; Proffitt et al., 2003). People can also perceive a target that is beyond arm’s reach to be closer when they intend to reach it with a tool (Witt et al., 2005; Osiurak et al., 2012). Taken together with our findings, this confirms that unconscious physiological states can play a key role even at so-called lower stages of perception and action realization. However, neurophysiological studies of thirst have mostly focused on the sensory aspects rather than on the behavioral implications of thirst (McKinley et al., 2019) and the neurophysiological bases of the effects of thirst on action structure remain to be explored more precisely.

## Ethics Statement

This study was carried out in accordance with the recommendations of local ethic committee of guidelines, Inserm Ethic Committee. The protocol was approved by the local ethic committee.

## Author Contributions

YR and FO contributed conception and design of the study. SC, ZB, and AI organized the database. PR, YR, and AF performed the statistical analysis. PR and YR wrote the first draft of the manuscript. SJ-C, DT, CN, MC, and FO revise it critically for intellectual content. All authors contributed to manuscript revision, read and approved the submitted version.

### Conflict of Interest Statement

The authors declare that the research was conducted in the absence of any commercial or financial relationships that could be construed as a potential conflict of interest.
